# Force Plate Assessment of Neuromuscular Jump Performance Under Loaded and Unloaded Conditions in Military Personnel

**DOI:** 10.3390/s26072217

**Published:** 2026-04-03

**Authors:** Julio A. Ceniza-Villacastín, Marcos A. Soriano, Diego A. Alonso-Aubín, Juan R. Godoy-López, Ester Jiménez-Ormeño

**Affiliations:** 1 Strength Training and Neuromuscular Performance Research Group (STreNgthP_RG), Faculty of Health Sciences HM Hospitals, University Camilo José Cela, 28692 Madrid, Spain; marcosa.soriano@urjc.es (M.A.S.); diegoalexandre.alonso@ucjc.edu (D.A.A.-A.); 2 Grupo de Pesquisa Atleta Tático (GPAT), Brazilian Army, Rio de Janeiro 22291-090, RJ, Brazil; 3 Center for Sport Studies, Rey Juan Carlos University, 28692 Madrid, Spain; 4 Directorate of Sport, Exercise and Physiotherapy, University of Salford, Frederick Road Campus, Manchester M6 6NY, UK; 5 Faculty of Health Sciences HM Hospitals, University Camilo José Cela, C/Castillo de Alarcón, 49, Villanueva de la Cañada, 28692 Madrid, Spain; 6 HM Hospitals Health Research Institute, 28015 Madrid, Spain; 7 Department of Training, Central School of Physical Education of the Army, 45006 Toledo, Spain; jgodoylo@et.mde.es; 8 Department of Physical Education, Sport and Human Movement, Universidad Autónoma de Madrid, 28049 Madrid, Spain

**Keywords:** countermovement jump, load carriage, tactical populations, sensor-based monitoring

## Abstract

**Highlights:**

**What are the main findings?**

- Loaded conditions induce a significant reduction in jump height and efficiency (RSI) across both slow and fast-SSC tasks in military personnel.
- During the CMJ, soldiers adopt a modified movement strategy characterized by prolonged phase durations and increased absolute force production, while maintaining a constant countermovement depth.
- In the CMRJ, decrements in force-time characteristics are primarily driven by increased ground contact time, with a limited capacity to increase force production relative to body mass to compensate for the demands imposed by loaded conditions.
- Metrics classified as force-based “drivers” maintain high reliability under loaded conditions, whereas fast-SSC rebound “outcome” metrics exhibit reduced reliability and greater variability.

**What are the implication of the main findings?**

- The CMJ represents a robust and highly reliable tool for monitoring neuromuscular readiness under loaded conditions in tactical populations.
- Training interventions for military personnel should target improvements in reactivity and force production under loaded conditions, to mitigate the performance decrements observed when operating with tactical equipment.
- Practitioners should exercise caution when interpreting CMRJ outcome metrics under loaded conditions due to increased trial-to-trial variability.

**Abstract:**

(1) Background: Military personnel are required to perform high-intensity actions and tactical tasks under external load, which increases system weight and alters movement mechanics. Understanding how these loaded conditions influence neuromuscular performance is essential for informing physical preparation and readiness monitoring. This study quantified the effects of tactical equipment on countermovement jump (CMJ) and countermovement rebound jump (CMRJ) force–time characteristics in active military personnel and evaluated the within-session reliability of these metrics under loaded and unloaded conditions; (2) Methods: Eighteen male soldiers performed CMJ and CMRJ assessments on dual force plates (1000 Hz) under unloaded and loaded conditions (standardized tactical equipment: 10.6 ± 1.18 kg). Force–time variables were categorized as strategy (phase durations, countermovement depth), driver (mean braking and propulsive force), and outcome (jump height, jump momentum, and modified reactive strength index; mRSI) metrics; (3) Results: CMJ outcome and driver metrics demonstrated good to excellent reliability under load (ICC ≥ 0.87; CV ≤ 8.4%), whereas CMRJ outcome variables showed reduced reliability and greater variability. Loaded conditions reduced jump height and mRSI in both CMJ and CMRJ (*p* < 0.05), while jump momentum and absolute mean force production increased, whereas force production relative to body mass decreased. During the CMJ (slow-SSC), participants exhibited longer braking and propulsive phase durations, indicating a temporal change in movement strategy under load, whereas CMRJ (fast-SSC) force–time characteristics showed increased contact time and reduced rebound metrics; (4) Conclusions: Overall, fast stretch–shortening cycle tasks appear more sensitive to loading conditions, whereas the CMJ provides a more robust and reliable assessment for monitoring neuromuscular performance in military personnel, particularly when considering both absolute and relative force responses.

## 1. Introduction

Military personnel are routinely required to perform demanding physical activities across diverse operational environments, including field deployments, urban operations, and obstacle course training [1,2]. These physical activities involve sprinting, climbing, crawling, jumping, and lifting or dragging injured teammates, frequently under loaded conditions imposed by tactical equipment such as ballistic vests, helmets, weapons, ammunition, and communication systems [3,4,5,6]. This equipment, essential for survival and mission success, substantially increases the mechanical and physiological demands placed on the musculoskeletal system [1,5,7,8].

Depending on mission type and duration, additional items such as hydration systems, rucksacks, or specialized tools may be added, elevating the total carried load to 30–45% of body mass [7]. Such loads are primarily distributed over the torso and upper limbs, shifting the center of mass forward, increasing trunk inclination, and altering locomotor mechanics to maintain balance and propulsion [7,9]. A high level of physical fitness is essential [10], as they must move their own body mass and equipment as efficiently as possible, resulting in physically repetitive actions under external load, which further modify movement kinematics, alter ground reaction forces, and heighten force demands during landing and deceleration [1,7,11,12]. These increases in external load contribute to substantial mechanical stress on the lower limb structures, helping explain the high rates of musculoskeletal injuries observed in military populations [12,13,14]. Therefore, understanding how external load influences lower-limb force-time characteristics is essential for informing physical preparation, tactical training, and deployment operations [5].

Previous research examining the effects of loaded conditions on physical and neuromuscular capacity in tactical populations has consistently reported performance decrements and altered biomechanical patterns [3,5]. Load carriage reduces mobility, running velocity, agility, and overall operational effectiveness [15]. Vertical jump height, for example, decreases by 8–9% when loads of 31 kg are applied, accompanied by a reduction of approximately 5% in vertical jump peak power [16]. Load carriage also increases by 30% completion time in simulated combat and tactical tasks when carrying 25–31 kg [3], and obstacle-navigation time can rise by 25% when loads increase from 14 to 27 kg, with total task duration increasing up to 47% in multi-obstacle courses [17,18]. Furthermore, external load reduces sprint performance, with 30 m sprint times increasing by 31.5% under a 21.6 kg load [18,19], and impairs agility, with obstacle course speed decreasing by 11–17% and successful obstacle negotiation dropping from 55% to 27% as load increases from 14 to 27 kg [15,20]. It should be noted that these external loads are typically reported as absolute masses, as tactical equipment configurations correspond to fixed-weight units that are standardized operationally, and military personnel are anthropometrically and physically conditioned to tolerate and perform under these non-relative load demands [21]. These decreases are accompanied by significant increases in ground reaction forces (GRF), with external load raising peak GRF by approximately 13–19% before exercise and by an additional 4–9% after prior running [3], and heavier loads also produce progressive increases in forefoot, midfoot, and hindfoot GRFs across both limbs [11,22].

Physical performance in military populations has traditionally been assessed using field tests based on repetitions or completion times, such as push-ups, sit-ups, and timed runs [23]. However, these assessments do not capture the underlying mechanical processes that lead to performance or injury risk [11,24,25], providing only a final outcome without describing how that outcome was produced [4]. To evaluate loaded conditions more comprehensively, military research has incorporated tests such as loaded marching and displacement [26], including endurance marches and treadmill protocols, walking on different surfaces [27], mobility tasks using specialized equipment [16,18]. Loaded jump and landing assessments [3,5], and loaded sprinting and agility tests [4,5,15] have been commonly employed to examine the effects of external load carriage. Among these tests, force plates remain the gold standard for quantifying kinetic variables such as ground reaction forces, rate of force development, impulse, and jump height, due to their high sensitivity, validity, and reproducibility across human performance contexts [11,28].

Jump protocols, and particularly the countermovement jump (CMJ), are extensively validated in sports science for assessing the lower limb’s stretch-shortening cycle (SSC) capacity. SSC tasks are commonly categorized according to ground contact time (GCT), distinguishing slow-SSC actions (≥~250 ms), such as the countermovement jump, from fast-SSC actions (≤~250 ms), typically assessed through rebound-type jumps such as the countermovement rebound jump (CMRJ) [29,30,31]. However, their application in tactical populations remains limited, despite the ability of force plate–derived metrics to provide objective and reliable insights into lower-limb force-time characteristics [11,29,32,33]. External load may influence these SSC regimes differently, as slow-SSC tasks allow greater time for force generation, whereas fast-SSC tasks require rapid force application within a constrained time window [29,34]. Moreover, a limited number of studies have examined how loaded conditions alter neuromuscular function using force plates, including the combined slow–SSC and fast–SSC responses under loaded conditions, using tests such as the CMJ and CMRJ, despite their direct relevance to tasks requiring rapid deceleration followed by explosive reacceleration [3,11]. This gap underscores the need to increase the number of studies focused on standardized force plate assessments to evaluate neuromuscular performance under loaded conditions in military personnel [33]. Therefore, the aim of the present study was to quantify the effects of tactical equipment on lower-limb force–time characteristics during the CMJ and CMRJ in active military personnel using force–time derived metrics. A secondary objective was to evaluate the within-session reliability of these force–time variables under both loaded and unloaded conditions to determine their suitability for monitoring lower-limb mechanical function in military operational and research contexts.

## 2. Materials and Methods

### 2.1. Study Design

A cross-sectional experimental design was employed in this study to assess the impact of loaded conditions on lower-limb force–time characteristics in military personnel.

**Figure 1:**
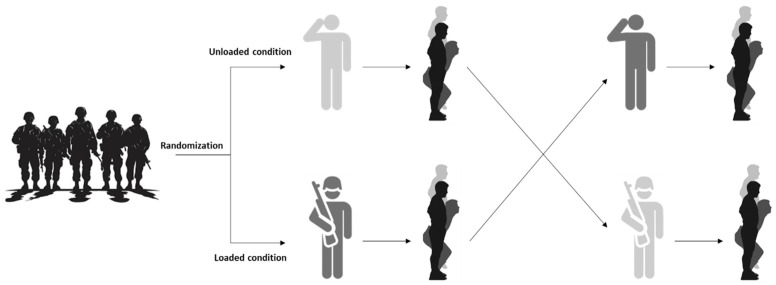
Experimental approach of the study.

For this purpose, participants were evaluated in a single testing session. The order of the testing conditions was randomized and counterbalanced across participants to minimize potential order effects. Each participant performed three trials of the CMJ and CMRJ tests under both loaded (with tactical equipment) and unloaded (with sportswear) conditions (Figure 1). Therefore, all participants completed both experimental conditions, acting as their own control within a within-subject crossover design. The tests were performed using a previously validated set of wireless dual force plates [11,30,35]. The randomizations were performed using a research randomizer software (www.randomizer.org).

### 2.2. Participants

An a priori sample size estimation for a matched pairs *t*-test using G Power software (version 3.1.9.6, Heinrich Heine University Düsseldorf, Düsseldorf, Germany) indicated that a sample size of 10 would be sufficient, considering a one-tailed test, a medium effect size (g = 0.9), an alpha level of 0.05, and a statistical power of 0.8. Eighteen healthy male soldiers from the officer and non-commissioned officer ranks of the Spanish Army (age = 39.61 ± 5.42 years; height = 1.78 ± 0.07 m; body mass = 76.04 ± 7.78 kg; body mass index = 24.13 ± 2.12 kg/m^2^) voluntarily participated in this study. Participants had more than 5 years of military experience, and all of them were free of musculoskeletal injuries in the three months preceding the investigation. Participants signed a written informed consent prior to their participation. All protocols were conducted in accordance with the principles outlined in the Declaration of Helsinki by the World Medical Association, and it was approved by the ethics review board of the University Camilo José Cela (code: 15_23_RNM_TA).

### 2.3. Procedures

The testing session was preceded by a 10-min standardized warm-up, which included general activation such as jogging and dynamic stretching, lower-limb strength exercises (e.g., squats, lunges, etc.), and neuromuscular activation exercises (e.g., submaximal CMJs, rebound jumps). Then, participants completed the jump tests in a randomized and counterbalanced order under both loaded and unloaded conditions [11,30,35].

#### 2.3.1. Loaded and Unloaded Conditions

**Figure 2:**
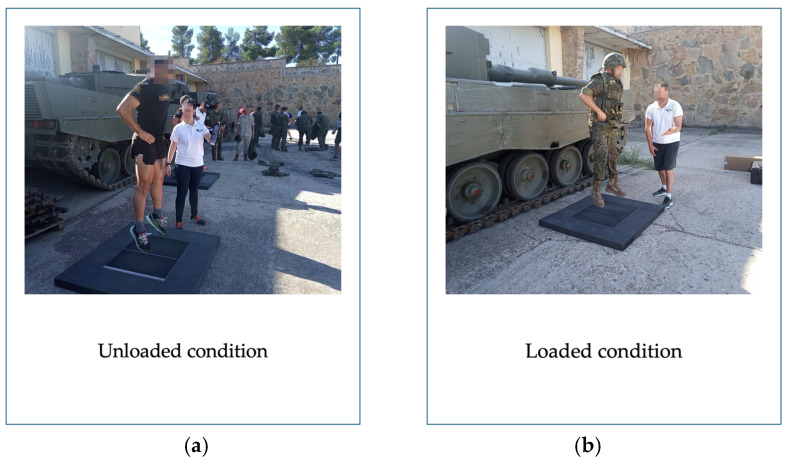
Unloaded and loaded conditions. (**a**) Unloaded condition (**left**): participants wearing standard sportswear; (**b**) Loaded condition (**right**): participants equipped with standardized tactical equipment, including helmet, boots, vest, and magazines.

Participants were evaluated in two conditions: loaded and unloaded (Figure 2). In the loaded condition, soldiers wore a standardized set of tactical equipment consisting of a helmet, vest, magazines, uniform and boots [36,37]. The core components were the same for all participants, but small variations in total mass are inherent to operational equipment. These variations arise primarily from individual sizing requirements such as larger or smaller uniform garments, boot sizes, helmet shell and padding configurations, which influence the total amount of material and, consequently, the mass of each item. Elements such as magazine pouches and uniform textiles may also differ slightly in mass depending on fit, length, or manufacturing tolerances [13,38,39,40,41]. Load carriage during operational duties depends on mission requirements, tactical roles, and task duration, therefore, the total load can vary substantially across real operational scenarios [5,7,42]. To ensure a controlled experimental condition, a standardized equipment configuration was used in the present study. This configuration corresponded to the equipment routinely worn by soldiers during institutional instruction and physical training sessions. For this reason, the external load imposed by the tactical equipment was not assumed but individually quantified. Each participant was weighed on the force plates in both unloaded and loaded conditions prior to testing. The unloaded condition corresponded to body mass measured while participants wore standardized physical training attire consisting of athletic footwear, a short-sleeved T-shirt, and shorts. The mass of individual equipment items was not measured separately. Instead, the total external load was quantified directly at the system level using force plate measurements of system weight. The added load was calculated as the difference between the two force-plate readings (system weight in loaded condition minus system weight in unloaded condition). This procedure ensured that the exact external load was determined for each soldier, capturing subject-specific variation despite the shared equipment configuration. Based on these individualized measurements, the tactical equipment increased system weight by 103.99 ± 11.62 N (10.6 ± 1.18 kg).

#### 2.3.2. Jump Assessment Protocols

The CMJ was performed from an upright standing position and involved a rapid downward countermovement through coordinated flexion of the hips, knees, and ankles, followed by a propulsive phase leading to take-off [34]. The mechanical characteristics and phase structure of the CMJ force–time curve have been extensively described in the previous literature [32,34]. The CMRJ combines the mechanical characteristics of the CMJ with a subsequent reactive rebound [43]. This task consists of two consecutive actions: an initial CMJ immediately followed by a reactive rebound jump (RJ) upon landing [30], enabling the assessment of the transition from a slow-SSC action to a fast-SSC action [44]. Force plates allow an equivalent phase-based analysis for both the initial CMJ and the subsequent RJ, enabling the calculation of rebound contact time, the Reactive Strength Index (RSI), and the distribution of braking and propulsive forces during the rebound [30,34]. Furthermore, this analysis provides detailed information on how athletes transition from a slow-SSC action (CMJ) to a fast-SSC action (RJ), revealing changes in movement strategy, force–time characteristics, limb asymmetries, and neuromuscular responsiveness [32,43].

In the present study, the mean CMJ height preceding the rebound was 0.29 ± 0.06 m in the unloaded condition and 0.23 ± 0.06 m in the loaded condition, providing contextual information regarding the average drop height from which the reactive action was initiated. This phase-specific analysis is particularly relevant for the present study, as external loading can influence both the magnitude and temporal organization of force production [34,45]. The ability of the force plates to quantify braking and propulsive mechanics, and force-generation strategies across consecutive efforts allows for a comprehensive evaluation of how tactical equipment modifies lower-limb force-time characteristics in the slow-SSC (CMJ) and fast-SSC (RJ) tasks [11,44].

In addition to the standardized warm-up, participants performed two sets of three submaximal CMJs to prepare for the CMJ test [30]. Three minutes of recovery were provided between the conclusion of the specific warm-up and the start of the tests. Participants stepped onto the force plates, which were placed on flat, level ground and calibrated (zeroed) before each trial. Participants stood completely upright and motionless for at least 1 s to determine system weight [30]. Then, maximal efforts commenced following signals from the Hawkin Dynamics app (version 9.6.1; Hawkin Dynamics Inc., Westbrook, ME, USA), which included a visual flash and an audible beep on the tablet. Participants performed the CMJ and CMRJ at their preferred countermovement depth, with hands placed on the hips (arms akimbo), and were instructed to “jump as fast and as high as possible” for the CMJ, whereas during the rebound phase of the CMRJ participants were additionally instructed to perform an immediate, fast, and explosive rebound jump, minimizing ground contact time while attempting to achieve maximal height in the second jump [30,46]. Three maximum effort trials were conducted for each jump, with approximately 60 s of rest between trials [45,47]. A trial was considered invalid if participants failed to maintain the required technique (e.g., loss of hands-on-hips position), initiated the movement before the start signal, performed an asymmetric take-off or stepping action on the force plates, or failed to execute the rebound immediately after landing in the CMRJ condition. In such cases, the attempt was discarded and repeated after the scheduled recovery period to obtain a valid trial. A maximum of two additional attempts was permitted if necessary to complete the three valid trials required for analysis.

Participants performed three CMJ trials, and three CMRJ trials in each condition (loaded and unloaded) to compare the effects of loaded conditions on jump performance. All assessments were conducted using the force plate system described in Section 2.3.3, under identical environmental and procedural conditions [30,35].

#### 2.3.3. Force Plate Equipment and Data Acquisition

The vertical component of the ground reaction force (vGRF) for all jump trials was collected using the same wireless dual force plate system described above (Hawkin Dynamics Inc., Westbrook, ME, USA), sampling at 1000 Hz and low-pass filtered at 50 Hz [30,35]. The Hawkin Dynamics software, running on a tablet device [Android operating system] and connected to the plates via Wi-Fi direct, was used to record and export the force–time data after each trial. System weight was first obtained in the unloaded condition following Hawkin’s Dynamics guidelines [30]. Participants stood motionless on the force plates for at least 1 s, and the mean vGRF during this quiet standing period was used as the unloaded system weight [34]. Body weight was then calculated by dividing this value by the acceleration due to gravity (9.81 m·s^−2^). In the loaded condition, the same procedure was repeated to obtain the loaded system weight; however, this value was not used to recalculate body weight. Instead, the external load associated with each participant’s tactical equipment was determined as the difference between the loaded and unloaded system weights. This condition-specific system weight (unloaded or loaded) was subsequently used for all force plate-derived calculations.

For the CMJ, the onset of movement was identified when vGRF deviated from system weight by more than a threshold of ±5 standard deviations (SD) of the force recorded during the 1 s quiet standing period, typically corresponding to the beginning of the unweighting phase [34,48]. Numerical integration of the net vGRF (vGRF minus system weight) began at the last value closest to system weight before movement onset, to ensure zero center-of-mass (COM) velocity at the start of integration [30,48]. Force–time metrics were then automatically calculated by the software using a forward-dynamics approach [30,34]. CMJ variables were selected following an Outcome–Driver–Strategy (ODS) framework to ensure a mechanistically coherent interpretation of performance [29,49]. Outcome metrics included jump height (m; calculated from take-off velocity), jump momentum (kg·m·s^−1^), and the modified reactive strength index (mRSI; arbitrary units; calculated as jump height divided by time to take-off) [30,50]. Driver metrics were selected to represent the mechanical determinants of jump performance and included mean propulsive force (N) and mean braking force (N) [29,30]. Strategy metrics were used to describe how these forces were applied and included time to take-off (s), countermovement depth (m), and braking phase duration (s) [29,34,50].

For the CMRJ, the force plates allowed separate analysis of the initial CMJ and the subsequent RJ. The first jump was processed using the same procedures described above for the CMJ [30,51]. For the rebound phase, the onset of ground contact was defined as the instant at which vGRF rose above a threshold based on the flight-phase noise (typically ≥5 SD of the flight signal), and the end of contact was identified when vGRF again fell below this threshold at take-off [30,51]. CMRJ variables were selected using the same ODS framework, with a specific focus on fast stretch-shortening cycle performance [43,49,52]. Outcome metrics included rebound jump height (m), rebound jump momentum (kg·m·s^−1^), and the reactive strength index (RSI; arbitrary units; calculated as rebound jump height divided by rebound contact time) [31,50]. Driver metrics included mean propulsive force (N) and mean braking force (N) during the rebound phase [31]. Strategy metrics included rebound contact time (s) and rebound countermovement depth (m), describing the temporal and displacement characteristics of the rebound action [29].

In addition to absolute force metrics, relative force variables were calculated by normalizing mean propulsive force and mean braking force to body mass (N·kg^−1^). Body mass was derived from force plate measurements of system weight obtained during the unloaded condition and was used to normalize the corresponding force variables in both unloaded and loaded conditions for the CMJ and CMRJ. For both CMJ and CMRJ, the average of the three recorded trials in each condition (loaded and unloaded) was used for the subsequent statistical analyses. Trials were visually monitored by the research team, and any attempt displaying a clear technical error (such as loss of balance, arm movement, or an improperly executed test) was discarded and repeated [46]. Trials were also repeated whenever jump height deviated by more than 10% from the participant’s preceding attempts [53,54]. Only valid trials were retained for analysis, ensuring that a minimum of three acceptable repetitions per condition were collected.

#### 2.3.4. Statistical Analyses

All data are presented as mean ± standard deviation. As jump height and braking phase duration in the CMJ violated the assumption of normality in both conditions, mean braking force under loaded conditions, and rebound jump height, rebound jump momentum, rebound countermovement depth, rebound contact time, and rebound mean propulsive force in the CMRJ violated normality in at least one condition according to the Shapiro–Wilk test (*p* < 0.05), a consistent non-parametric approach was adopted for all paired comparisons to ensure methodological consistency across variables, given the small sample size and the presence of non-normal distributions in multiple metrics.

The reliability of CMJ and CMRJ metrics across the three trials within each condition (loaded and unloaded) was evaluated using intraclass correlation coefficients (ICC, model 3.1) with associated 95% confidence intervals (CI). ICC values were interpreted according to Koo and Li [55]: <0.50 = poor, 0.50–0.75 = moderate, 0.75–0.90 = good, and >0.90 = excellent reliability. Absolute reliability was examined using the coefficient of variation (CV), with values ≤10% considered acceptable [56,57]. The standard error of measurement (SEM) and its 95% CI were calculated following Weir [58], using the formula SEM = SD [pooled] × √((1 − ICC)). The smallest detectable difference (SDD) was calculated as SDD = 1.96 × (√2]) × SEM. All reliability computations were performed using a custom Microsoft Excel spreadsheet based on the analytical framework described by Hopkins [54,59]. Comparisons between unloaded and loaded conditions for all CMJ and CMRJ variables were conducted using the Wilcoxon signed-rank test. This decision was based on the presence of non-normally distributed variables and the paired nature of the experimental design, ensuring an appropriate within-subject comparison while maintaining a consistent statistical approach across mechanical outcomes. Effect sizes were calculated as Cohen’s d for paired samples to quantify the magnitude of within-subject changes, independent of the inferential test used, and interpreted as trivial (<0.20), small (0.20–0.59), moderate (0.60–1.19), large (1.20–2.00), very large (2.00–4.00), and extremely large (>4.00) [59]. All statistical analyses were performed using JASP statistical software (version 0.17.3, Amsterdam, The Netherlands). Statistical significance was set at *p* ≤ 0.05.

## 3. Results

### 3.1. Reliability for the CMJ Tests

Across both unloaded and loaded conditions, CMJ outcome and driver metrics demonstrated good to excellent reliability, with ICC values generally ranging from 0.87 to 1.00, CV values ranging from 0.79 to 9.71% in the unloaded condition and from 1.8 to 17% in the loaded condition, and consistently low SEM and SDD values relative to the magnitude of the measured variables, remaining within or close to the commonly accepted threshold of 10% variability for reliable performance measures, as recommended by Hopkins [59]. Strategy metrics showed slightly greater variability, with time to take-off and countermovement depth presenting moderate to good reliability (ICC = 0.84–0.88), while braking phase duration exhibited moderate reliability (ICC = 0.75) and higher CV values (~10%) (Table 1).

In the loaded condition, a similar reliability pattern was observed. Outcome metrics retained good to excellent reliability (ICC = 0.93–0.99; CV = 1.8–8.1%), and driver metrics again demonstrated good reliability, with ICC values ranging from 0.87 to 0.95. Strategy metrics showed increased variability under load, particularly for braking phase duration, which displayed lower reliability (ICC = 0.63) and higher CV values (~17%). Time to take-off and countermovement depth maintained moderate reliability (ICC = 0.81–0.82) (Table 2).

### 3.2. Reliability for the CMRJ Tests

In the unloaded condition, CMRJ outcome metrics demonstrated good reliability, with rebound jump height, rebound jump momentum, and rebound RSI showing ICC values ranging from 0.83 to 0.93. Variability remained acceptable for rebound jump height and momentum (CV = 3.6–7.9%), whereas rebound RSI exhibited slightly higher variability (CV ≈ 11.7%), which is commonly observed for ratio-based fast stretch-shortening cycle indices (Table 3). Driver metrics during the rebound phase, including mean propulsive force and mean braking force, showed good to excellent reliability (ICC = 0.77–0.90) with moderate variability (CV = 6.6–9.6%). Strategy metrics, such as rebound countermovement depth and rebound contact time, demonstrated moderate to good reliability (ICC = 0.82–0.83), although greater variability was observed for countermovement depth.

Under loaded conditions, the overall reliability of CMRJ metrics was lower compared with the unloaded condition, with outcome variables demonstrating poor to moderate reliability (ICC < 0.60). Rebound jump height and rebound jump momentum exhibited lower reliability (ICC = 0.48–0.54) and higher variability (CV up to 18.9%), indicating greater trial-to-trial dispersion under loaded conditions. In contrast, rebound RSI retained moderate reliability (ICC = 0.73; CV ≈ 12%) under loaded conditions (Table 4). Rebound driver metrics maintained moderate to good reliability under loaded conditions, with ICC values ranging from 0.69 to 0.82, although variability increased slightly compared with the unloaded condition. Strategy metrics showed a similar pattern, with rebound contact time demonstrating good reliability (ICC = 0.84) despite increased variability (CV ≈ 11.6%), while rebound countermovement depth exhibited moderate reliability (ICC = 0.65).

### 3.3. Comparison Between Unloaded and Loaded CMJ Conditions

Jump height and mRSI were substantially higher in the unloaded condition (both *p* < 0.001; large effects, d = 0.97–0.98). In contrast, jump momentum was slightly but significantly greater in the loaded condition (*p* = 0.044; small effect, d = −0.32) (Table 5). Mean propulsive force and mean braking force were significantly higher in the loaded condition (both *p* < 0.001; moderate effects, d = −0.57 to −0.62). Time to take-off and braking phase duration were significantly longer in the loaded condition (*p* = 0.005 for both; small effects, d = −0.43 to −0.44). Countermovement depth did not differ between conditions (*p* = 0.49; trivial effect; d = −0.11).

### 3.4. Comparison Between Unloaded and Loaded CMRJ Conditions

For the CMRJ, loaded conditions significantly impaired key fast-SSC outcome metrics. Rebound jump height and rebound RSI were higher in the unloaded condition (both *p* < 0.001; large and moderate effects, d = 0.93 and 0.77, respectively) (Table 6). Rebound jump momentum did not differ between conditions (*p* = 0.231; trivial effect, d = −0.188). In contrast to the CMJ, rebound driver metrics were not significantly affected by loaded condition. Mean propulsive force and mean braking force during the rebound phase showed no clear differences between unloaded and loaded conditions (*p* = 0.212 and *p* = 0.698, trivial effects, d = 0.212 and 0.698, respectively). Rebound contact time was significantly longer under loaded conditions (*p* = 0.011; small effect, d = −0.40), whereas rebound countermovement depth did not differ between conditions (*p* = 0.593; trivial effect, d = −0.084).

## 4. Discussion

The present study quantified the effects of tactical equipment on neuromuscular performance and force–time characteristics during slow–SSC (CMJ) and fast–SSC (CMRJ) tasks, and evaluated the within-session reliability of these metrics in military personnel [60]. Given the increasing integration of force plate monitoring within military training environments [11], establishing the reproducibility of these metrics under loading conditions is essential before interpreting alterations in force–time characteristics [28]. First, the CMJ demonstrated high-to-excellent reliability across unloaded and loaded conditions, supporting its suitability for neuromuscular readiness monitoring under external load [60,61]. In contrast, CMRJ outcome metrics exhibited reduced reliability when performed under loaded conditions, indicating higher trial-to-trial variability during fast-SSC actions [31]. Second, loaded conditions were associated with reduced jump performance and altered mechanical responses, although the pattern of adaptation differed between CMJ and CMRJ tasks [3,42]. The inclusion of both absolute and relative force metrics allowed a more precise interpretation of these adaptations, highlighting that increases in absolute force did not necessarily reflect an improved capacity to generate force relative to body mass. While CMJ force-application capacity is sustained through a prolonged temporal strategy under loaded conditions, CMRJ efficiency decreases due to an inability to increase force production within the limited contact time characteristic of the fast-SSC [3,62], accompanied by a marked reduction in the reliability of rebound outcome metrics [29,60].

### 4.1. Reliability of Force-Plate Metrics in Loaded and Unloaded Conditions

From a monitoring perspective, the CMJ demonstrated high-to-excellent reliability (ICC = 0.81–0.99) for most outcome and driver metrics across both conditions, supporting its utility as a robust tool for neuromuscular readiness assessment in military settings [48]. However, braking phase duration exhibited higher variability under loaded conditions (CV ≈ 17%; ICC = 0.63), and this elevated variability in phase-based temporal variables suggests that small observed changes should be interpreted cautiously, as they may reflect increased movement variability under load rather than true neuromuscular fatigue [61]. In contrast, the CMRJ demonstrated reduced reliability under loaded conditions. While reliability was good in the unloaded condition, rebound jump height and momentum ICCs dropped to ~0.50 when loaded. This aligns with Smith et al. (2023), who reported that reactive metrics in military personnel can exhibit low stability (ICC as low as 0.28) due to the interference of tactical equipment [60]. The movement of the vest, helmet, and the individualized distribution of tactical equipment may have contributed to greater variability in COM behavior during rapid rebound phases. Additionally, fast-SSC tasks such as the CMRJ are inherently more sensitive to small perturbations in body configuration and external load distribution, which may amplify measurement variability when tactical equipment is worn due to load-induced alterations in postural control and movement patterns [60,63]. Greater variability does not necessitate the exclusion of specific metrics, but rather requires interpretation within their measurement-error boundaries [29,55]. In the present study, CMJ outcome and driver metrics retained low variability under loaded conditions (ICC = 0.93–0.99; CV = 1.8–8.1%), with SEM and SDD values remaining within acceptable thresholds [50,54]. In contrast, CMRJ outcome metrics showed reduced reliability when loaded (ICC = 0.48–0.54; CV up to 18.9%), reflecting greater dispersion during fast-SSC actions [60]. Practitioners should therefore interpret longitudinal changes in loaded rebound metrics relative to SEM and SDD thresholds to determine whether observed alterations exceed expected measurement noise, rather than dismissing these variables solely because of their greater variability [58].

### 4.2. Effects of External Load on CMJ Performance

In the CMJ, the addition of external load (~10.6 kg) resulted in significant reductions in jump height (−20.3%) and mRSI, consistent with prior research in tactical populations [3]. However, the analysis of strategy metrics reveals that this reduction cannot be explained solely by the increase in system mass, but also reflects a shift toward a force-oriented, time-dependent strategy [5,29,34]. Participants exhibited significant increases in time to take-off and braking phase duration, suggesting a need to prolong the impulse generation period to overcome the inertia associated with loaded conditions [3,5]. This lengthening of the braking phase reflects a shift toward a slower SSC profile, likely to maximize force development under higher mechanical demand [31,34]. A key finding was the maintenance of countermovement depth across conditions (trivial effect). This indicates that military personnel did not alter their displacement strategy to increase the work distance, potentially due to the physical restrictions imposed by the ballistic vest or a stiffening strategy to maintain stability [7,64]. To compensate, participants exhibited higher absolute mean propulsive and braking forces; however, when expressed relative to body mass, these force metrics were reduced under loaded conditions, indicating that the increase in absolute force did not fully compensate for the greater mechanical demand imposed by the additional load [5]. This distinction between absolute and relative force responses is critical, as it suggests that although participants increased total force output, their capacity to generate force relative to their body mass was diminished under load [3,16]. Interestingly, jump momentum increased under loaded conditions, confirming that the increase in system mass dominated the momentum equation despite the prolonged time to take-off and reduced jump height [50,51,53]. These results align with Ladlow et al. (2025) regarding the mechanical cost of vertical propulsion under loaded conditions and underscore that such conditions reduce movement efficiency by necessitating a more time-dependent neuromuscular solution [28].

### 4.3. Effects of External Load on CMRJ Performance

The impact of loaded conditions on the fast-SSC (CMRJ) resulted in a more pronounced degradation of reactive efficiency. Unlike the CMJ, in which force output increased to accommodate the additional demands, mean rebound forces expressed in absolute terms did not differ between conditions, whereas the relative rebound force metrics were lower under loaded conditions. This indicates that during fast–SSC actions, where ground contact times are constrained, participants are unable to sufficiently scale force output relative to body mass to meet the increased mechanical demands imposed by loaded conditions [3,11]. Consequently, decrements in jump force–time metrics were primarily driven by a significant increase in rebound contact time and a moderate reduction in RSI [3,65]. Furthermore, rebound jump momentum did not differ between conditions (trivial effect) [3,11]. These findings suggest a near-complete loss of mechanical advantage, whereby take-off velocity decreased in direct proportion to the increase in system mass, indicating that loaded conditions constrain rapid force–velocity expression during reactive tasks [51,53]. This pattern, in which absolute force output is largely preserved while relative rebound force and reactive performance are reduced, suggests that loaded conditions disproportionately penalize high-velocity, reactive movements compared with slower strength–power tasks [3,66].

### 4.4. Practical Applications and Limitations

The findings have direct implications for the physical preparation and neuromuscular performance monitoring of military personnel [11,29]. The phase-based analytical framework adopted in the present study (strategy–driver–outcome classification) provides practitioners with a structured approach to interpret force–time data beyond isolated outcome measures, allowing the identification of how force–time characteristics are generated and how they are altered under external load. This approach facilitates the distinction between reductions in outcome performance attributable to increased system mass and those resulting from modified force–time strategies under loaded conditions [5].

First, the reduction in reactive efficiency (RSI) and increased contact times under loaded conditions suggest that training programs should not only focus on absolute strength but also target the preservation of fast-SSC capabilities while operating under external load [3]. Accordingly, training programs should incorporate controlled exposure to loaded plyometric and ballistic tasks performed with tactical equipment, with the aim of maintaining rapid force application and minimizing contact time despite increased system mass [67].

Second, the prolongation of braking and propulsive phase durations during the CMJ demonstrates that soldiers adopt a temporally modified movement strategy to accommodate increased system mass [11]. Monitoring both unloaded and loaded conditions is therefore recommended, as comparing these states allows practitioners to detect load-induced shifts in force–time characteristics, identify compensatory strategies, and quantify the additional mechanical demands imposed by tactical equipment [5]. Such comparisons may also help identify individuals who rely predominantly on temporal prolongation and increased absolute force production, but who are less able to preserve force output relative to body mass when performing under load [66].

Third, given the increased mechanical stress and elevated ground reaction forces associated with external load carriage, integrating force plate monitoring into military training environments may support injury-risk mitigation by identifying individuals who exhibit excessive temporal prolongation, limited force production capacity, or marked decrements in fast-SSC performance under load [68]. Interventions aimed at enhancing force production capacity and the ability to apply force rapidly under loaded conditions may therefore assist in preserving performance while limiting excessive mechanical demand during repeated tasks performed with tactical equipment [5,69]. The CMJ should be prioritized for routine readiness monitoring due to its superior reliability under loaded conditions, particularly when driver metrics are used to evaluate force production capacity independently of changes in system mass [60].

Several limitations must be acknowledged. The sample consisted exclusively of male officers and non-commissioned officers, and the findings may not be generalizable to female personnel or individuals with different training backgrounds or experience levels (42). Additionally, the study utilized a fixed tactical equipment load (~10 kg); heavier loads (e.g., 30–45 kg rucksacks) may induce greater displacement strategies (e.g., increased countermovement depth), further temporal prolongation, or more pronounced alterations in force–time characteristics [8,62]. However, the fact that measurable changes in force–time characteristics were already observed under this load suggests that even relatively small increases in system mass may influence neuromuscular strategy during explosive tasks [3,5]. Moreover, the load used in the present study corresponds to the tactical equipment configuration routinely worn during training activities and physical conditioning sessions, which supports the ecological validity of evaluating its effects on neuromuscular performance [11].

A further limitation is the absence of maximal strength assessments (e.g., one-repetition maximum or isometric peak force), which precludes interpretation of how individual relative strength levels influenced the capacity to manage increased system mass [28]. Without quantification of maximal force-producing capacity, it is not possible to determine whether the observed temporal adaptations under load reflect strength limitations or task-specific mechanical adjustments [52]. Including maximal strength profiling in future research would allow clearer understanding of whether stronger individuals are better able to preserve force application rates and reactive performance under loaded conditions [16,41]. Future research should investigate the longitudinal effects of loaded conditions or targeted training interventions on mitigating performance decrements and improving the ability to sustain neuromuscular efficiency under operational loading demands.

## 5. Conclusions

The present study demonstrates that loaded conditions significantly alter neuromuscular strategy and are associated with reduced explosive performance in military personnel. During slow-SSC tasks (CMJ), the neuromuscular system compensates for the increased system mass by prolonging temporal phases and increasing absolute force production, although at the cost of reduced efficiency (mRSI) and performance (jump height). However, when expressed relative to body mass, force production is reduced under loaded conditions, indicating an incomplete mechanical compensation. In contrast, fast-SSC tasks (CMRJ) appear more susceptible to loading, as evidenced by increased contact times and a reduced ability to generate force relative to body mass within constrained time intervals. Furthermore, these findings highlight the critical importance of selecting reliable metrics for readiness monitoring.

## Figures and Tables

**Table 1:** Reliability of CMJ variables in the unloaded condition.

Variable	Mean ± SD	ICC (95% CI)	CV (%)	SEM	SDD
**Outcome metrics**					
Jump height (m)	0.31 ± 0.06	0.99 (0.98–1.00)	1.7	0.01	0.03
Jump momentum (kg·m/s)	189.68 ± 27.51	1.00 (0.99–1.00)	0.79	1.5	4.16
mRSI (a.u.)	0.36 ± 0.10	0.94 (0.84–0.98)	7.12	0.03	0.08
**Driver metrics**					
Mean propulsive force (N)	1441.34 ± 193.65	0.97 (0.92–0.99)	2.44	35.19	97.53
Mean braking force (N)	1232.63 ± 191.25	0.95 (0.87–0.98)	3.66	45.14	125.10
**Strategy metrics**					
Time to take off (s)	0.87 ± 0.14	0.84 (0.61–0.94)	7.01	0.06	0.17
Countermovement depth (m)	−0.32 ± 0.05	0.88 (0.70–0.95)	−6.58	0.02	0.06
Braking duration phase (s)	0.19 ± 0.03	0.75 (0.46–0.90)	9.71	0.02	0.06

Values are mean ± standard deviation (SD) across trials; ICC = intraclass correlation coefficient; CI = confidence interval; CV = coefficient of variation; SEM = standard error of measurement; SDD = smallest detectable difference; mRSI = modified reactive strength index.

**Table 2:** Reliability of CMJ variables in the loaded condition.

Variable	Mean ± SD	ICC (95% CI)	CV (%)	SEM	SDD
**Outcome metrics**					
Jump height (m)	0.24 ± 0.05	0.98 (0.94–0.99)	3.57	0.01	0.03
Jump momentum (kg·m/s)	192.81 ± 28.54	0.99 (0.96–0.99)	1.84	3.54	9.81
mRSI (a.u.)	0.27 ± 0.08	0.93 (0.81–0.97)	8.14	0.02	0.06
**Driver metrics**					
Mean propulsive force (N)	1516.97 ± 191.57	0.95 (0.88–0.98)	2.94	44.59	123.58
Mean braking force (N)	1316.51 ± 202.61	0.87 (0.67–0.95)	5.98	78.74	218.22
**Strategy metrics**					
Time to take off (s)	0.92 ± 0.13	0.81 (0.55–0.92)	6.01	0.06	0.17
Countermovement depth (m)	−0.31 ± 0.05	0.82 (0.57–0.93)	−7.48	0.02	0.06
Braking duration phase (s)	0.22 ± 0.06	0.63 (0.23–0.85)	16.53	0.04	0.11

Values are mean ± standard deviation (SD) across trials; ICC = intraclass correlation coefficient; CI = confidence interval; CV = coefficient of variation; SEM = standard error of measurement; SDD = smallest detectable difference; mRSI = modified reactive strength index.

**Table 3:** Reliability of CMRJ variables in the unloaded condition.

Variable	Mean ± SD	ICC (95% CI)	CV (%)	SEM	SDD
**Outcome metrics**					
Rebound jump height (m)	0.29 ± 0.05	0.83 (0.60–0.93)	7.9	0.02	0.06
Rebound jump momentum (kg·m/s)	184.19 ± 22.86	0.93 (0.83–0.97)	3.57	6.58	18.24
Rebound RSI (a.u.)	1.62 ± 0.44	0.83 (0.61–0.93)	11.72	0.19	0.53
**Driver metrics**					
Rebound mean propulsive force (N)	1871.34 ± 358.11	0.90 (0.75–0.96)	6.56	122.78	340.28
Rebound mean braking force (N)	2204.37 ± 423.55	0.77 (0.49–0.91)	9.59	211.39	585.85
**Strategy metrics**					
Rebound countermovement depth (m)	−0.18 ± 0.08	0.82 (0.58–0.93)	−19.34	0.03	0.08
Rebound contact time (s)	325.33 ± 90.69	0.83 (0.60–0.93)	11.82	38.44	106.53

Values are mean ± standard deviation (SD) across trials; ICC = intraclass correlation coefficient; CI = confidence interval; CV = coefficient of variation; SEM = standard error of measurement; SDD = smallest detectable difference; RSI = reactive strength index.

**Table 4:** Reliability of CMRJ variables in the loaded condition.

Variable	Mean ± SD	ICC (95% CI)	CV (%)	SEM	SDD
**Outcome metrics**					
Rebound jump height (m)	0.23 ± 0.06	0.54 (0.12–0.80)	18.86	0.04	0.11
Rebound jump momentum (kg·m/s)	184.80 ± 34.02	0.48 (0.03–0.77)	14.83	27.41	75.97
Rebound RSI (a.u.)	1.35 ± 0.32	0.73 (0.41–0.89)	11.96	0.16	0.44
**Driver metrics**					
Rebound mean propulsive force (N)	1907.03 ± 299.38	0.82 (0.57–0.93)	7.24	138	382.46
Rebound mean braking force (N)	2190.42 ± 383.43	0.69 (0.34–0.87)	10.02	219.53	608.41
**Strategy metrics**					
Rebound countermovement depth (m)	−0.18 ± 0.09	0.65 (0.28–0.85)	−32.09	0.06	0.17
Rebound contact time (s)	349.65 ± 98.74	0.84 (0.63–0.94)	11.61	40.6	112.52

Values are mean ± standard deviation (SD) across trials; ICC = intraclass correlation coefficient; CI = confidence interval; CV = coefficient of variation; SEM = standard error of measurement; SDD = smallest detectable difference; RSI = reactive strength index.

**Table 5:** Comparison between unloaded and loaded CMJ conditions.

Variable	Unloaded (Mean ± SD)	Loaded (Mean ± SD)	*p*-Value	Effect Size (Cohen’s d, 95% CI)	Interpretation	Direction
**Outcome metrics**						
Jump height (m)	0.306 ± 0.056	0.244 ± 0.052	<0.001	0.979 [0.961 to 0.989]	Moderate	Unloaded ↑
Jump momentum (kg·m/s)	189.677 ± 27.012	192.811 ± 27.988	0.044	−0.318 [−0.564 to −0.020]	Small	Loaded ↑
Modified RSI (a.u.)	0.364 ± 0.103	0.274 ± 0.078	<0.001	0.965 [0.936 to 0.981]	Moderate	Unloaded ↑
**Driver metrics**						
Mean propulsive force (N)	1441.34 ± 193.65	1516.97 ± 191.57	<0.001	−0.620 [−0.776 to −0.393]	Moderate	Loaded ↑
Relative mean propulsive force (N/kg)	18.54 ± 1.52	17.09 ± 1.40	<0.001	0.912 [0.842 to 0.952]	Moderate	Unloaded ↑
Mean braking force (N)	1232.63 ± 191.25	1316.51 ± 202.61	<0.001	−0.574 [−0.745 to −0.331]	Small	Loaded ↑
Relative mean braking force (N/kg)	15.83 ± 1.84	14.80 ± 1.51	<0.001	0.630 [0.406 to 0.782]	Moderate	Unloaded ↑
**Strategy metrics**						
Time to take off (s)	0.869 ± 0.136	0.916 ± 0.130	0.005	−0.437 [−0.651 to −0.157]	Small	Loaded ↑
CMJ depth (m)	−0.322 ± 0.054	−0.313 ± 0.053	0.49	−0.110 [−0.397 to 0.197]	Trivial	No clear difference
Braking phase (s)	0.194 ± 0.034	0.216 ± 0.057	0.005	−0.443 [−0.656 to −0.165]	Small	Loaded ↑

Values are mean ± standard deviation (SD); CI = confidence interval; mRSI = modified reactive strength index. ↑ indicates higher values in the indicated condition (unloaded or loaded).

**Table 6:** Comparison between unloaded and loaded CMRJ conditions.

Variable	Unloaded (Mean ± SD)	Loaded (Mean ± SD)	*p*-Value	Effect Size (Cohen’s d, 95% CI)	Interpretation	Direction
**Outcome metrics**						
Rebound jump height (m)	0.288 ± 0.051	0.227 ± 0.056	<0.001	0.929 [0.872 to 0.961]	Moderate	Unloaded ↑
Rebound jump momentum (kg·m/s)	184.193 ± 22.448	184.801 ± 33.739	0.231	−0.188 [−0.460 to 0.116]	Trivial	No clear difference
Rebound RSI (a.u.)	1.623 ± 0.435	1.351 ± 0.313	<0.001	0.772 [0.617 to 0.870]	Moderate	Unloaded ↑
**Driver metrics**						
Rebound mean propulsive force (N)	1871.34 ± 358.11	1907.03 ± 299.38	0.212	−0.196 [−0.466 to 0.108]	Trivial	No clear difference
Relative rebound mean propulsive force (N/kg)	24.06 ± 4.05	21.59 ± 3.13	<0.001	0.813 [0.680 to 0.894]	Moderate	Unloaded ↑
Rebound mean braking force (N)	2204.37 ± 423.55	2190.42 ± 383.43	0.698	0.061 [−0.240 to 0.352]	Trivial	No clear difference
Relative rebound mean braking force (N/kg)	28.40 ± 5.29	21.59 ± 3.13	<0.001	0.997 [0.995 to 0.999]	Moderate	Unloaded ↑
**Strategy metrics**						
Rebound countermovement depth (m)	−0.18 ± 0.08	−0.18 ± 0.09	0.593	−0.084 [−0.372 to 0.219]	Trivial	No clear difference
Rebound contact time (s)	325.333 ± 89.325	349.648 ± 97.157	0.011	−0.404 [−0.628 to −0.118]	Small	Loaded ↑

Values are mean ± standard deviation (SD); CI = confidence interval; RSI = reactive strength index. ↑ indicates higher values in the indicated condition (unloaded or loaded).

## Data Availability

The data presented in this study are available on request from the corresponding authors. The data are not publicly available due to privacy and institutional restrictions related to the nature of the participating military personnel.

## Abbreviations

The following abbreviations are used in this manuscript:

GRF	Ground Reaction Force
vGRF	vertical Ground Reaction Force
SSC	Stretch-Shortening Cycle
CMJ	Countermovement Jump
CMRJ	Countermovement And Rebound Jump
RJ	Rebound Jump
GCT	Ground Contact Time
ODS	Outcome-Driver-Strategy
RSI	Reactive Strength Index
mRSI	Modified Reactive Strength Index
SD	Standard Deviation
COM	Center of Mass
CI	Confidence Intervals
ICC	Intraclass Correlation Coefficients
CV	Coefficient of Variation
SEM	Standard Error of Measurement
SDD	Smallest Detectable Difference
